# Leukemia in India: Insights Into Incidence, Prevalence, Mortality, and Disability-Adjusted Life Years

**DOI:** 10.7759/cureus.62557

**Published:** 2024-06-17

**Authors:** Dinesh N Nalage, P. S Kudnar, Rahul Langhe, Balasaheb Ughade, Tejswini Sontakke, Ashwini Biradar

**Affiliations:** 1 Department of Molecular Biology and Microbiology, Institute of Biosciences and Technology, MGM University, Aurangabad, IND; 2 Department of Zoology, Postgraduate Research Center, Modern College of Arts, Science, and Commerce (Autonomous), Pune, IND; 3 Department of Botany, Amdar Shashikant Shinde Mahavidyalaya, Medha, IND; 4 Department of Zoology, Mahilaratna Pushpatai Hiray (MPH) Arts, Science and Commerce Mahila Mahavidyalaya, Mahatma Gandhi Vidyamandir (MGV), Malegaon, IND; 5 Department of Microbiology, Sub-Campus Osmanabad, Dr. Babasaheb Ambedkar Marathwada University, Osmanabad, IND

**Keywords:** india, risk factor., disability-adjusted life years (dalys), malignancies, leukemia, cancers

## Abstract

Objective: This study aimed to examine the impact of leukemia and other cancers in India and to observe any changes over time.

Methodology: Detailed estimates of incidence, prevalence, mortality, and disability-adjusted life years (DALYs) for 30 types of cancers in India were analyzed for 29 years from 1990 to 2019 as part of the Global Burden of Diseases (GBD) study. Data from all available sources were used to gather information on the overall burden of disease in India.

Results: Cancer is a leading cause of death worldwide, with varying rates of incidence in India, making prevention and treatment a challenge. Because cancer is not a reportable disease in India, the overall burden estimate is still a work in progress. This study analyzed the impact of leukemia and other cancers in India, including trends in incidence, DALYs, and mortality related to all cancers and various malignancies. The causes of leukemia in India were also explored.

Conclusions: The study found the trends of cancer types that account for the majority of leukemia-related and cancer-related DALYs, death, prevalence, and incidence in India. Among the four most frequent malignancies, such as leukemia, there was significant variation based on age. Over the last 29 years, mortality from chronic myelogenous leukemia (CML) and acute lymphocytic leukemia (ALL) has decreased, while deaths from acute myelogenous leukemia (AML) and chronic lymphocytic leukemia (CLL) have increased steadily.

## Introduction

Cancer is the world's second greatest cause of mortality after cardiovascular disease [1-3]. In 2020-2021, the COVID-19 pandemic significantly impacted cancer detection and treatment. Healthcare institution closures resulted in reduced access to care and a short-term decline in cancer and leukemia incidence [4]. However, this was followed by an increase in advanced-stage sickness and ultimately higher mortality rates.

Patients with cancer in low- and middle-income countries, such as India, often have a worse prognosis than those in high-income countries due to low cancer awareness, late detection of leukemia, and limited or unequal access to affordable treatment options [5]. With 1.3 billion individuals dispersed throughout numerous territories and states, India has a heterogeneous distribution of leukemia due to varying levels of growth, and genetic variation in populations, habitats, and lifestyles [6].

Previous studies have attempted to explain cancer burden and epidemiological patterns in various regions of India, but systematic and complete knowledge of the volume and temporal trends of all malignancies in India remains inadequate. Given that healthcare delivery in India is a government responsibility, this information is critical in guiding actions for cancer reduction that meet the needs of the population [7-9].

The India State-Level Illness Burden Initiative and the Global Burden of Leukemia Injuries and Risk Factors Study joined forces to create disease burden estimates at a subnational level for India. Using data from the Global Burden of Disease (GBD), they recently published variations in health status across Indian states from 1990 to 2016. In 2016, it was estimated that 10,500 children (ages 0-14) and 5,090 teenagers (ages 15-19) would be diagnosed with cancer, with 1,190 and 590 dying from the disease, respectively. However, these estimates exclude benign and borderline malignant brain tumors as they were not mandatory to be reported to cancer registries until 2004 and are based on 15 years of historical incidence data. The most frequent kind of cancer in children is leukemia, accounting for 28% of cases. Brain and other nervous system malignancies come in second with 27% of instances, with over 25% of these being benign/borderline malignant. This study offers a thorough summary of each form of cancer and leukemia incidence and health-related mortality trends in India from 1990 to 2019.

## Materials and methods

Using the terms "cancer," "burden," "leukemia," "death," "cause of death," "DALY," "incidence," "epidemiology," "India," "morbidity," "neoplasm," "mortality," "trends," and "prevalence," we explored PubMed and currently accessible reports in 2019 for assessments of cancer load across India. There were no language or publication date restrictions. The DALYs, mortality, incidence, and prevalence of all cancer and leukemia types in India throughout time were not properly defined in any article we found, although there is a lot of important information on the distribution of cancer in India.

The GBD Injuries and Risk Factors Study 2019 included the study as a component and analyzed data from all accessible sources to examine all causes of sickness burden. To the best of our knowledge, this study is the first to offer precise estimates of DALYs, death, incidence, and prevalence for 30 different types of malignancies across 29 years from 1990 to 2019.

## Results

This study was part of the GBD 2019, which investigated every cause of disease burden using information obtained from numerous available resources. Although the age-standardized prevalence of every kind of cancer evaluated collectively has not changed significantly in India over the last 29 years, cancer's share of the total burden of diseases has more than doubled according to this study. This study emphasizes the large differences between Indian states in terms of cancer-related mortality, incidence, and disability-adjusted life years (DALYs), as well as the fact that tobacco use is the country's biggest cause of cancer.

Overall status of cancer in India

**Figure 1:**
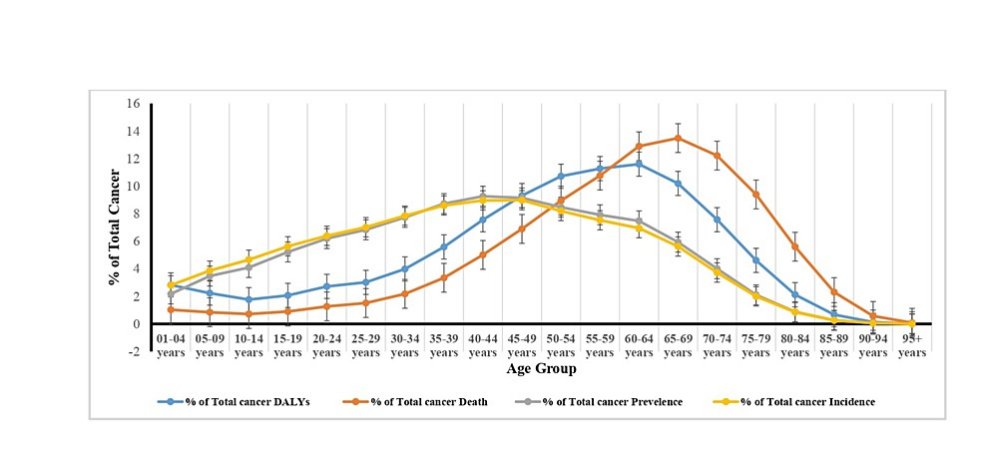
Percentage of total cancer DALYs, death, prevalence, and incidence of different types of cancers with age group in India, 2019. DALY, disability-adjusted life year

In India, the prevalence of all cancers increased by 138% and 118%, respectively, since 1990, which accounts for 5.83% (5.12%-6.69%) of total DALYs and 9.93% (9.26%-10.68%) of all fatalities. Between 1990 and 2019, there was a rise in the prevalence of all types of cancer, rising from 0.61% or 2.02% (1.66%-2.47%) to 2.63% (2.16%-3.16%) of all prevalent cases. The incidence of all types of cancer has increased from the 0.24% recorded in 1990 to up to 0.36% of all new cases in 2019. From 1990 to 2019, the total death rate percentage and DALYs percentage of all cancers with respect to age will exponentially increase up to the age of 65-69 years and 60-64 years from birth and decline after the age of 70 and 65 years, respectively. However, the total incidence percentage and total prevalence percentage will exponentially increase up to the age of 40-49 years from birth and decline after the age of 50 years (Figure 1).

**Figure 2:**
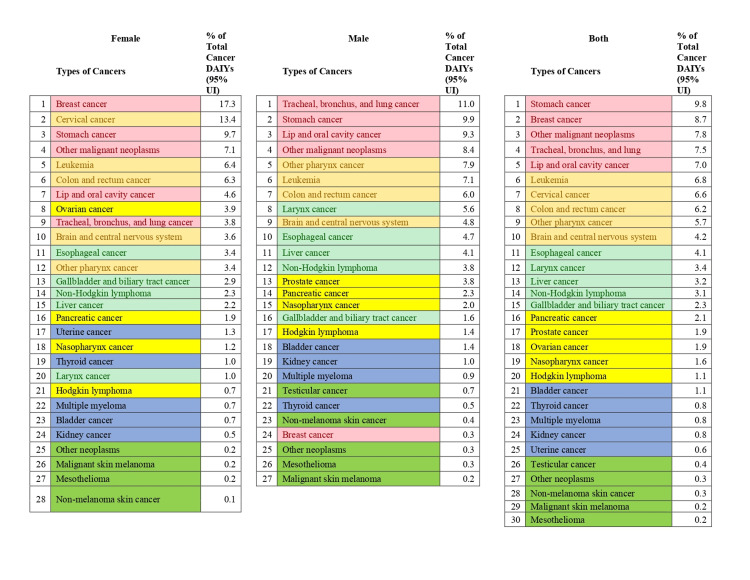
Percentage of total cancer DALYs due to different types of cancers by sex in India, 2019. The types of cancers are color-coded in groups based on their ranking in both sexes combined (pink indicates 1 to 5 ranking, faint yellow indicates 6 to 10 ranking, faint green indicates 11 to 15 ranking, dark yellow indicates 16 to 20 ranking, blue indicates 21 to 25 ranking, and dark green indicates 26 to 30 ranking). DALY, disability-adjusted life year

In 2019, the most common types of cancer were stomach cancer (9.8%); breast cancer (8.7%); other malignant neoplasms (7.8%); tracheal, bronchus, and lung cancer (7.5%); lip and oral cavity cancer (7.0%); leukemia (6.8%); cervical cancer (6.6%); colon and rectum cancer (6.2%); and other pharynx cancer (5.7%). These diseases also accounted for more than 5.8% of the total cancer DALYs (Figure 2).

Leukemia

When the DNA in leukocytes, a kind of blood cell, becomes mutated or altered, it loses its ability to govern growth and division, resulting in leukemia. These altered cells may be able to elude the immune system and grow uncontrolled, displacing healthy cells in the process. Chronic lymphocytic leukemia (CLL), chronic myelogenous leukemia (CML), acute lymphocytic leukemia (ALL), and acute myelogenous leukemia (AML) are the four primary kinds of leukemia distinguished by the afflicted cell type (myeloid or lymphoid) and progression rate (chronic or acute). Although the global incidence of leukemia is largely steady, there are regional variabilities related to ethnicity, environment, and lifestyle factors. The specific etiology of leukemia is uncertain; however, radiation exposure is one of the potential risk factors. In this study, we looked at four variables: DALYs, deaths, prevalence, and incidence.

DALYs

**Figure 3:**
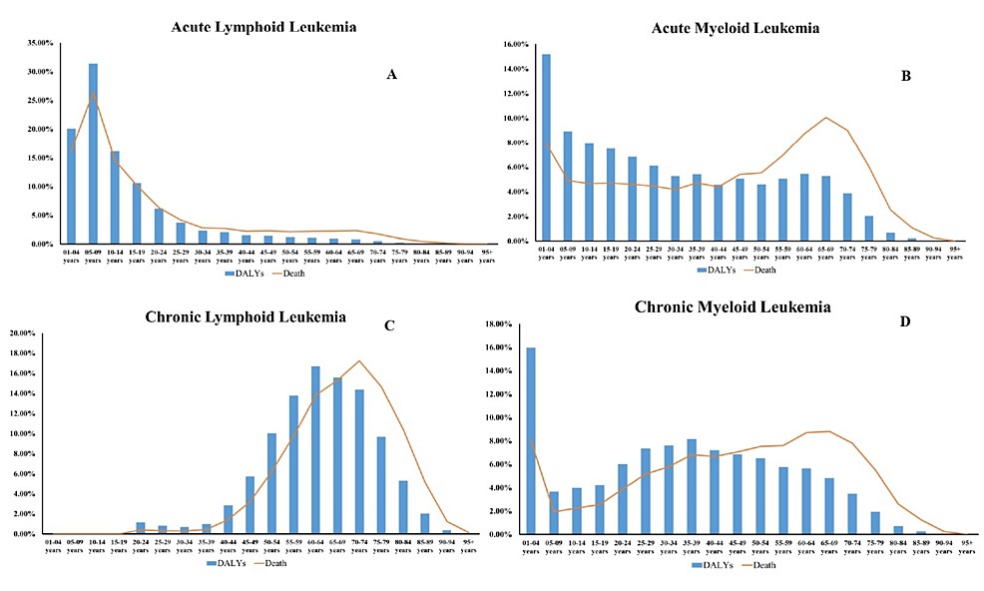
Comparison of DALYs and death of leukemia subtypes of all age groups with total percentage: (A) acute lymphoid leukemia, (B) acute myeloid leukemia, (C) chronic lymphoid leukemia, and (D) chronic myeloid leukemia.

From 1990 to 2019, leukemia ranked sixth in terms of DALYs among all malignancies, accounting for around 6.8% of all cancers. Males are more prone than females to get leukemia, with a 0.7% greater prevalence in males. Leukemia accounts for 7.1% of all malignancies in men and 6.4% in women (Figure 2). We discovered that the percentage of CLL and AML grew considerably over 1990 and 2019, but the percentage of CML, ALL, and other kinds of leukemia declined in both males and females. In 2019, AML contributed to more than 10.5% of all leukemia cases across both sexes. CLL was found to be more prevalent in females in 2019, with a rate of 12.48% compared to 2.85% in males. In females in 2019, the percentage of ALL was 18.22%, CML 21.34%, and other leukemia 14.26%, showing a decreasing trend compared to the 1990s. Meanwhile, in males in 2019, ALL was 24.63%, CML 26.68%, and other leukemia 15.91%, also showing a declining trend compared to the 1990s (Figure 3).

Prevalence

Leukemia is the sixth most prevalent malignancy between 1990 and 2019 (excluding other neoplasms), making up around 6.79% of all malignancies. With a difference of 0.78%, men are more likely than women to get leukemia. Leukemia is more frequent in men (7.17%) than in women (6.39%).

Similar tendencies to those in the DALYs and mortality measures were seen in the examination of the prevalence of leukemia subtypes. Males and females both had an average prevalence of AML in 2019 of 27.90%. With a frequency of 18.22% in females and 4.85% in males, CLL is more common in females than in males. In females, the prevalence of ALL, CML, and other leukemia shows a negative trend compared to the 1990s, with percentages of 18.22%, 21.34%, and 14.26%, respectively. However, in males, the prevalence of ALL, CML, and other leukemia also shows a negative trend compared to the 1990s, with percentages of 24.63%, 26.68%, and 15.91%, respectively.

Incidence

Leukemia ranks 6th in incidence among all cancers (excluding other neoplasms) from 1990 to 2019, accounting for 4.83% of total cancers. Males are more likely to have leukemia than females, with a 2.24% higher incidence in males. The incidence of leukemia in males was 6.27%, whereas 4.03% in females.

An analysis of leukemia subtypes in terms of incidence shows a pattern similar to the findings from DALYs, death, and prevalence measures. Males saw an average AML incidence that was somewhat greater than females in 2019. CLL had a higher incidence in females at 29.19% compared to 7.76% in males. ALL was the number one cause of DALYs and deaths in India in 2019 for both boys and girls aged 0 to 20. In both sexes aged 0-30 and 60-75 years, AML was the largest cause of DALYs and the top cause of mortality. In both males and females aged 55 to 75 years , CLL was the largest cause of DALYs, and in people aged 60 to 80 years, it was the main cause of mortality. CML was the leading cause of DALYs and death in both males and females aged 0-4 years, with a uniform distribution of cases in other age groups (Figure 3).

Death

Leukemia ranked ninth in terms of deaths among all cancers from 1990 to 2019, accounting for approximately 4.6% of all cancers. Males are more likely to die from leukemia than females, with a 0.5% higher incidence in males. In males, leukemia causes 4.84% of all cancer deaths, while in females it causes 4.34%.

**Figure 4:**
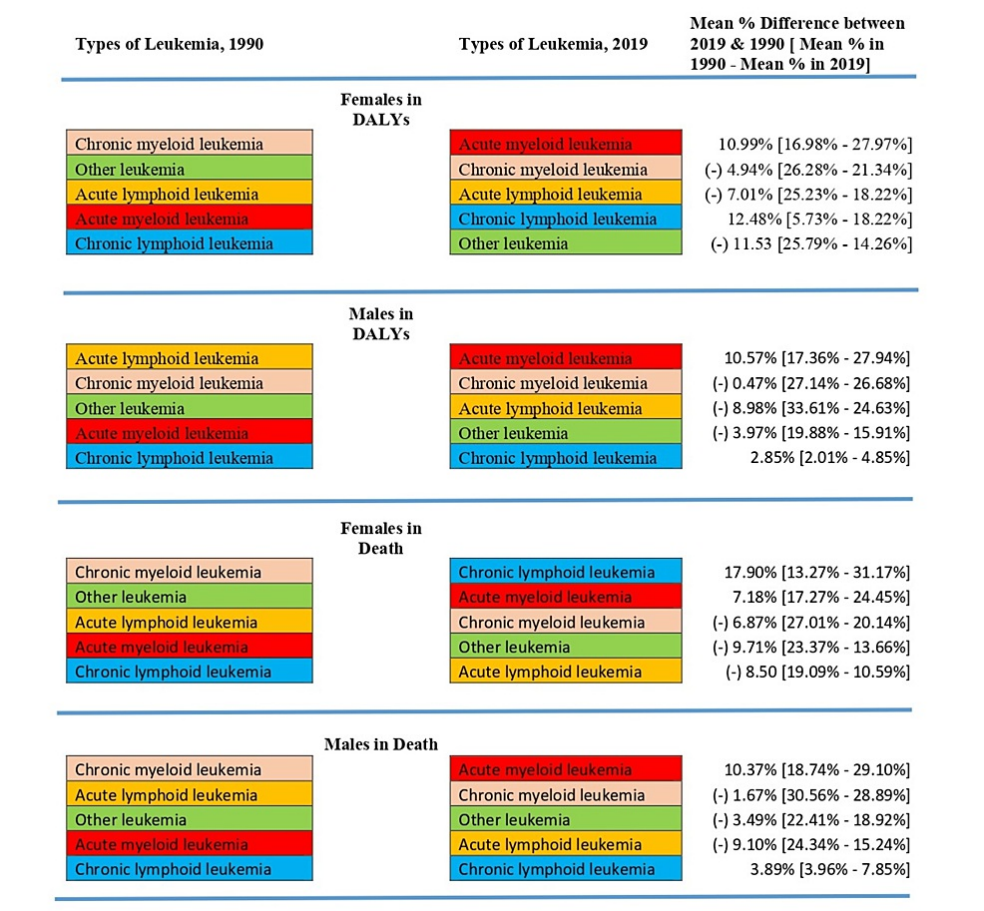
Change in DALYs and death in males and females for different types of leukemia in India, 1990-2019. Faint pink indicates chronic myeloid leukemia, red indicates acute myeloid leukemia, faint yellow indicates acute lymphoid leukemia, blue indicates chronic lymphoid leukemia, and green indicates other leukemia. DALY, disability-adjusted life year

The subtype analysis of deaths from leukemia mirrors the results seen in the DALYs analysis. The proportion of AML and CLL cases grew significantly between 1990 and 2019, but the proportion of ALL, CML, and other kinds of leukemia declined in both sexes. In terms of fatalities among males in 2019, AML was the most common subtype. Women's deaths from CLL accounted for 17.90% of all deaths in 2019, compared to men's deaths from CLL which accounted for 3.89% of all deaths. In 2019, the proportion of mortality in females due to ALL was 10.59%, CML was 20.14%, and other leukemia was 13.66%, showing a decline from the 1990s. In contrast, ALL caused 15.24% of fatalities in men in 2019, CML caused 28.89%, and other leukemia caused 18.92%, all of which are on the decline compared to the 1990s (Figure 4).

## Discussion

From 1990 to 2019, new cases of cancer and its fatalities in India increased, as did cancer's proportionate contribution to total DALYs and fatalities in the nation. Over 29 years, all-cause cancer DALYs, mortality, and incidence increased significantly. The age-standardized incidences in both sexes combined, however, did not change, highlighting the contribution of population growth and aging to the nation's expanding cancer load [10]. During this time, the age-standardized cancer and leukemia mortality rate rose for men, indicating gender inequalities. In every state of the nation, men had greater mortality index (MI) ratios than females.

The observed patterns of cancer type and sex-specific occurrence prevalence rates in India over the years may be due to aging, advancements in cancer knowledge, assessment, health treatment, and various risk factors. Nationwide age-standardized prevalence rates of stomach cancer have dramatically declined, which may be attributable to alterations in lifestyle, such as eating less food stored with salt, having greater access to refrigeration, eating more fruit, and stopping smoking [11,12]. Since the infection with *Helicobacter pylori* remains widespread in Indians, this is unlikely to be the reason why the incidence of stomach cancer has reduced [13]. In India, lower parity, age of birth, and an expansion in obesity and overweight are a few examples of the specific risk factors that have changed over time and are significantly linked to a rise in the age-standardized prevalence rate of breast cancer [14-16]. The nation's stagnation may be caused by a mixture of mixed trends in India's causes, such as a decrease in indoor air pollution and smoking but a rise in outdoor pollution, as well as patterns of additional unexplained risk factors. A minor drop was observed in the age-standardized lung cancer incidence rate [14,17]. Also, the occurrence rate of oral cavity and lip malignancy in India over the last decade might be attributed to a drop in the usage of smokeless tobacco in the country. The fertility risks for breast cancer development discussed above may have a negative correlation with the falling age-standardized incidence rate of cervical cancer [15]. Due to a lack of frequent human papillomavirus (HPV) testing, there is no indication of growing seroprevalence of HPV and its variants across a period in India. Decomposition studies are needed to disentangle the contributions of community structure changes, lifestyle factors, therapies, and other variables to the patterns of India's major malignancies. The occurrence of various types of cancer varies greatly across India.

The increase in AML and CLL in the elderly population can be attributed to genetic and epigenetic changes, cumulative environmental exposures, and immune system decline. Also, the elderly population is influenced by age-related changes in the microbiome [18-22], which can impact immune function and inflammation, contributing to cancer development. Aging leads to the accumulation of mutations and chronic inflammation, while lifestyle factors and comorbid conditions further elevate the risk. Additionally, hematopoietic stem cell aging contributes to the higher incidence of these leukemias. Overall, these multifactorial influences result in the observed rise in AML and CLL among older adults.

Although leukemias are connected to infectious, genetic, and environmental hazards, the reasons for India's age-standardized incidence rate's considerable reduction from 1990 to 2019 remain unexplained. We identified a new pattern of rapid growth in CLL and myeloid leukemia, but not other cancers, in the aging Indian population. In a study published after 2011, Hao et al. discovered the major aging-dependent leukemia trend changes [23]. Therefore, the majority of the impact of the World Health Organization (WHO) revisions has already been taken into account. Numerous intrinsic variables contribute to hematopoietic stem cell (HSC) aging, putting committed myeloid progenitor cells and HSC at risk for genetic and epigenetic alterations, the emergence of pre-leukemic clones, and eventually malignant transformation. This may aid in comprehending why age has a greater influence on the incidence of CML and AML. CLL is a disease of differentiated mature B cells that affects the elderly as opposed to myeloid leukemia. The age-related accumulation of epigenetic and genetic modifications that support clonal B-cell proliferation is the basis for the molecular pathogenesis of CLL. Surprisingly, recent research found that in CLL patients, the ability to create clonal B cells had already been gained at the HSC stage. As a result, the beginning and development of CLL may be influenced by HSC aging. In the future, we anticipate that developing novel strategies to stop or decrease HSC aging will help with the diagnosis, prognosis, and therapy of myeloid leukemia and CLL. More concerted efforts will be needed in the future to better comprehend HSC aging mechanisms and find novel leukemia prevention strategies.

This systematic assessment and comprehensive analysis of the disparities in the prevalence and patterns of cancer types across India may be useful for future cancer care and preventive planning. Additionally, cancer registry coverage in rural regions and several big Indian states that do not presently have registries should improve. To understand the causes of the shifting patterns of various kinds of malignancies in India, further large-scale collaborative research is required.

## Conclusions

Ultimately, this study's detailed etiology of 30 forms of cancer in India over the past 29 years highlights the stark differences in leukemia rates for each type of cancer, and it can be used as a useful guide for more precise cancer control planning that considers the patterns of various cancers in India. The rising cancer rate in India should spur the development of more systematic and extensive approaches to lowering the disease's effects on the populace. These measures should include enhanced human resources and infrastructure for cancer and leukemia prophylaxis, screening, medication and palliative care, and appropriate financial support for cancer treatment. The 10 malignancies that cause the most DALYs in India - breast, stomach, lung, oral cavity and lip, pharynx, colon and rectum, leukemia, cervical, etc. - should be the focus of these new techniques to overcome the burden of leukemia cases. Myeloid leukemia and CLL cases grew quickly, with a clear association with population aging. Although the entire impact of the aging of the *baby boomers* on cancer occurrences would not be known for another decade, the growth in myeloid leukemia and CLL looked to outstrip that of other malignancies. As conditions and resources permit, other forms of cancer should be treated in India.

From 1990 to 2019, this research presents complete descriptive epidemiology of leukemia, other cancers, and its heterogeneity across all Indians. This is our brief overview of the responsible factors for leukemia and other cancer cases in India.

## References

[1] Demarinis S (2020). Cancer overtakes cardiovascular disease as leading cause of death in wealthy nations. Explore (NY).

[2] Kocarnik JM, Compton K, Dean FE (2022). Cancer incidence, mortality, years of life lost, years lived with disability, and disability-adjusted life years for 29 cancer groups from 2010 to 2019: a systematic analysis for the Global Burden of Disease Study 2019. JAMA Oncol.

[3] Fitzmaurice C, Akinyemiju TF, Al Lami FH (2018). Global, regional, and national cancer incidence, mortality, years of life lost, years lived with disability, and disability-adjusted life-years for 29 cancer groups, 1990 to 2016: a systematic analysis for the Global Burden of Disease study. JAMA Oncol.

[4] Siegel RL, Miller KD, Fuchs HE, Jemal A (2021). Cancer Statistics, 2021. CA Cancer J Clin.

[5] Molyneux E, Israels T, Howard SC (2020). Cancer Treatment in Low- and Middle-Income Countries. Oxford Textbook of Cancer in Children.

[6] (2018). The burden of cancers and their variations across the states of India: the Global Burden of Disease Study 1990-2016. Lancet Oncol.

[7] Basu A, Kumar J, Barua A (2022). Surgical Oncology, All India Institute of Medical Sciences, Department of Surgical Oncology, India: evolution and recent advances in MIS in head and neck cancers: a comprehensive review. J Cancer Res Rev Rep.

[8] Chaturvedi M, Sathishkumar K, Lakshminarayana SK, Nath A, Das P, Mathur P (2022). Women cancers in India: Incidence, trends and their clinical extent from the National Cancer Registry Programme. Cancer Epidemiol.

[9] Coelho KR (2012). Challenges of the oral cancer burden in India. J Cancer Epidemiol.

[10] Fitzmaurice C, Allen C, Barber RM (2017). Global, regional, and national cancer incidence, mortality, years of life lost, years lived with disability, and disability-adjusted life-years for 32 cancer groups, 1990 to 2015: a systematic analysis for the Global Burden of Disease study. JAMA Oncol.

[11] Lynn BD, Figueroa J, Biritwum R (2020). Breast cancer age-specific incidence rates among Ghanaian women by breast cancer risk factors: a study using census and population-based case-control study data. Cancer Res.

[12] Zaheer S, Shah N, Maqbool SA, Soomro NM (2019). Estimates of past and future time trends in age-specific breast cancer incidence among women in Karachi, Pakistan: 2004-2025. BMC Public Health.

[13] Khasag O, Boldbaatar G, Tegshee T (2018). The prevalence of Helicobacter pylori infection and other risk factors among Mongolian dyspeptic patients who have a high incidence and mortality rate of gastric cancer. Gut Pathog.

[14] (2020). Indian Council of Medical Research, Public Health Foundation of India, Institute for Health Metrics and Evaluation: GBD India Compare Data Visualization. New Delhi.

[15] Dhillon PK, Yeole BB, Dikshit R, Kurkure AP, Bray F (2011). Trends in breast, ovarian and cervical cancer incidence in Mumbai, India over a 30-year period, 1976-2005: an age-period-cohort analysis. Br J Cancer.

[16] (2018). The increasing burden of diabetes and variations among the states of India: the Global Burden of Disease Study 1990-2016. Lancet Glob Health.

[17] (2018). The changing patterns of cardiovascular diseases and their risk factors in the states of India: the Global Burden of Disease Study 1990-2016. Lancet Glob Health.

[18] Nalage D, Sontakke T, Biradar A (2023). The impact of environmental toxins on the animal gut microbiome and their potential to contribute to disease. Food Chem Adv.

[19] Patil R, Satpute R, Nalage D (2023). The application of omics technologies to toxicology. Toxicol Adv.

[20] Sontakke T, Biradar A, Dixit P (2022). Metagenomics and microbiome of infant: old and recent instincts. Microenviron Microecol Res.

[21] Patil R, Sontakke T, Biradar A (2023). Zinc: an essential trace element for human health and beyond. Food Health.

[22] Nalage D, Sontakke T, Patil R (2022). First Three Days of Life. First Three Days of Life.

[23] Hao T, Li-Talley M, Buck A, Chen W (2019). An emerging trend of rapid increase of leukemia but not all cancers in the aging population in the United States. Sci Rep.

